# ADHD and ASD traits are differentially associated with orientation sensitivity in a non-clinical adult sample

**DOI:** 10.3389/fpsyg.2025.1632880

**Published:** 2025-09-26

**Authors:** Vesko Varbanov, Paul G. Overton, Tom Stafford

**Affiliations:** Department of Psychology, The University of Sheffield, Sheffield, United Kingdom

**Keywords:** ADHD, autism, sensory processing, anxiety, neuro developemental disorders

## Abstract

**Objectives:**

Research indicates that ADHD and ASD are associated with sensory processing difficulties. However, psychophysical testing of this has primarily focused on ASD with no equivalent research on ADHD. The relationship between ADHD, ASD and sensory processing may also be influenced by anxiety. This study investigates whether orientation discrimination performance is differentially related to ADHD and ASD *traits* in a non-clinical adult sample, and whether anxiety statistically explains these associations.

**Methods:**

We measure visual orientation discrimination thresholds using a method of constant stimuli in a two-alternative forced choice paradigm with an adaptive, randomly interleaved procedure and a one up three down design. The task results are compared to reported trait expressions of ADHD, ASD and anxiety via correlational analysis. Following on this we conduct a mediation analysis to assess the possible mediating role of anxiety.

**Results:**

The ADHD and ASD trait expressions were associated with similar sensory processing abnormalities. The panic and generalized anxiety traits were only specifically associated with the ADHD-Hyperactive type and respective sensory thresholds. Such effects were not observed for any ASD traits.

**Conclusions:**

These findings suggest that while both ADHD and ASD traits are linked to reduced orientation sensitivity, only ADHD traits—particularly hyperactivity—show specific associations mediated by anxiety. This points to distinct underlying mechanisms in the sensory processing profiles of ADHD and ASD, with anxiety playing a more prominent role in ADHD-related impairments.

## Introduction

In accordance with the Diagnostic and Statistical Manual of Mental Disorders (5th ed.; DSM-5; American Psychiatric, Association, 2013) Attention Deficit/Hyperactivity Disorder (ADHD) is characterized by a dysfunction within the attention domain and excessive motor behavior manifested by hyperactivity and impulsivity. Its description thus differs from that of Autistic Spectrum Disorder (ASD), which the DSM-5 defines as a condition in which deficits fall within any of three domains—language ability, repetitive and rigid behavior, and social interactions (American Psychiatric Association, 2000; American Psychiatric, Association, 2013). These seemingly different manifestations suggest distinct conditions and indeed until the 5th edition of the DSM they could not be diagnosed together (American Psychiatric Association, 1994; Ramtekkar, 2017). However, as the two have similar genetic profile (Rommelse et al., 2010), research in the last decade has focused on their co-existence and has suggested that they not only intertwine (Hayashi et al., 2022) but also exacerbate each other (Rao and Landa, 2014; Gnanavel et al., 2019; Al-Beltagi, 2021). The question concerning their commonality is pressing now more than ever as more people experiencing difficulties within the academic, occupational and social domains in life are subsequently diagnosed with (either of) the two conditions (Lau-Zhu et al., 2019) and especially so in adult populations, where, research has thus far been scarce (Coghill and Sonuga-Barke, 2012; Kern et al., 2015).

Within the two conditions, reports have suggested similar difficulties exist in the registration, modulation and consecutive internal organization of sensory stimuli (Miller et al., 2007). This can lead to atypical (hyper or hypo) sensitivity across all sensory modalities (Jones et al., 2003; Rogers et al., 2003) and can result in difficulties meeting situational demands and being able to engage in daily activities (Lane and Reynolds, 2019; Ghanizadeh, 2011) as well as increased anxiety. This raises the question whether, in spite of their apparent differences, ADHD and ASD might come from a common neural substrate linked in some way to sensory processing (Panagiotidi et al., 2017a,b, 2019; Lane et al., 2011; Dellapiazza et al., 2021). Despite these sensory similarities the two conditions often exhibit distinct behavioral responses to sensory inputs. Similar sensitivities across the tactile, olfactory and auditory modalities can trigger aggressive behavior in ADHD individuals, while leading to social withdrawal in ASD, in both cases to some extent aided by anxiety, adding an additional layer of complexity to understanding how close or not they really are (Ghanizadeh, 2011).

It is noteworthy that pre-peri and postnatal factors, such as preterm birth, lead to different structural anomalies in ADHD and ASD, such as consistently decreased cortical thickness in ADHD (Narr et al., 2010; Hoogman et al., 2017) but variable levels of thickness in ASD (Khundrakpam et al., 2017; Sparks et al., 2002), delayed brain maturation in ADHD (Berger et al., 2013), but region-dependent maturation in ASD (O'Hearn and Lynn, 2023; Sparks et al., 2002), and overall increased brain volume in ASD but decreased brain volume in ADHD (Stevens and Haney-Caron, 2012). These neurodevelopmental differences may underlie distinct pathophysiological mechanisms in ADHD and ASD, adding complexity to the debate about their overlap or divergence. They are also reflected in divergent cognitive functions, including executive functioning, attention regulation, language, and visuospatial processing. For example, individuals with ADHD frequently display impairments in sustained attention and response inhibition, which have been linked to abnormalities in the superior colliculus (Overton, 2008; Krauzlis et al., 2013). In contrast, individuals with ASD often show heightened sensitivity to visual stimuli (Samson et al., 2012) and a tendency toward local over global processing (Schulz et al., 2023; Hubel and Wiesel, 1968; Haupt and Huber, 2008; London et al., 2013). These cognitive differences manifest behaviorally in tasks such as visual detection, visual search, and responses to embedded figures, where individuals with ASD have been previously reported to outperform those with ADHD (Almeida et al., 2010, 2012; Grinter et al., 2009), highlighting distinct processing profiles within the visual cognitive domain (Schulz et al., 2023; Chung and Son, 2020).

However, in contrast to the afore discussed differences in pathology, studies focused on subcortical brain structures have also reported similar abnormalities in various areas involved in the deployment of attentional resources, (hyperactive and/or repetitive) motor movements and problems in social interaction (Overton, 2008; Jure, 2022; Panagiotidi et al., 2017a; Luders et al., 2016; Lau et al., 2013). These findings thus suggest the conditions are more similar than not, but how these commonalities translate to similarities and differences in sensory processing between the two conditions remains insufficiently explored as perceptual processing studies, specifically with regards to (low level) visual processing, have largely concentrated on ASD groups and omitted ADHD groups, although the latter is more frequently diagnosed than the former (Polanczyk et al., 2007). Previous studies have also never compared (low level) visual processing in both conditions within the same cohort.

One crucial perceptual ability in humans- Visual Orientation Discrimination (VOD) is the ability to accurately perceive and differentiate between different orientations of sensory stimuli, such as visual patterns. It has been found that for the general neurotypical population oblique angles of presentation are more difficult to identify than vertical angles (Scobey, 1982; Taylor and Rodriguez, 2025). Some studies on VOD have suggested that individuals with high self-reported ASD rates find it easier to identify oblique angles than neurotypical individuals (Bertone et al., 2005) while others have yielded conflicting results with no difference between the ASD and neurotypical populations (Brock et al., 2011). However, no study has thus far compared ASD with ADHD or other comorbid conditions within the same cohort and in fact most studies, such as that of Dickinson et al. (2014), have only assessed within a single ASD group. A comparison of sensory behavior between ADHD and ASD within the same group is imperative in order to understand their possible connections.

In light of the contradicting results regarding the relationship between VOD and ASD traits, Dickinson et al. (2014) employed a highly sensitive task introducing an oblique as well as cardinal angle in order to ascertain if variability in VOD is associated with the level of ASD traits. They found superior visual performance in ASD, however failed to consider whether this performance was the result of pure enhancement of low level perception or other factors and further failed to discuss the confounding factors discussed above, such as comorbidities, which can interfere in task performance. The idea that comorbidities can play a role in ADHD/ASD behavior is not investigated enough despite other research suggesting up to 87% overlap between ADHD and ASD (Scandurra et al., 2019; Leitner, 2014; Leyfer et al., 2006). Thus the question to what extent these results would be similar or dissimilar, or influenced, by ADHD traits remains unanswered (Salunkhe et al., 2019). Hence a task for the current study is to compare the behavior of the two conditions within the same cohort and look at the role of comorbid traits.

In addition, as well as the need to investigate whether ADHD and ASD are similar or dissimilar in low level visual processing, the factors that may lead to such possible differences also need to be examined. As mentioned above, structural and functional research has demonstrated a relationship between abnormal sensory processing and heightened anxiety across various disorders, including ADHD and ASD (McMahon et al., 2019). Indeed, self-reported anxiety rates have been linked to heightened sensory processing mechanisms in the general population (Kinnealey and Fuiek, 1999) and replicated in clinical studies on generalized anxiety (GAD) (Xiao et al., 2011). Varbanov et al. (2023) reported that in their cohort anxiety had the properties of a key connecting and modulating factor between two clusters of inter and intrapersonal symptoms characteristic of ADHD and ASD and played a crucial role for the interaction between other comorbidities and the conditions. This further highlights the possibility that comorbidities contribute meaningfully to how ADHD and ASD functionally manifest. Additionally, because anxiety itself relies on sensory input and shows a strong comorbid connection with both ADHD and ASD (Degnan and Fox, 2007), it represents a particularly useful factor for investigating whether and how these conditions differ in terms of sensory processing.

To investigate the possible separation of ADHD and ASD in sensory processing, we measured sensory thresholds obtained from a VOD task using a method of constant stimuli. The pre-consciously inferred task results can provide reliable data on sensory processing which we then compare to reports on variations in ADHD, ASD trait expressions, and three types of anxiety—social, panic, and generalized. In our previous study (Varbanov et al., 2023), we identified a significant role of anxiety in the relationship between sensory sensitivity and neurodevelopmental traits, prompting the present investigation into its potential mediating function. The inclusion of panic anxiety and GAD in the current models is further supported by extensive literature showing anxiety as a frequent comorbidity in ADHD and ASD, particularly in individuals with heightened sensory responsiveness (e.g., Green et al., 2012; Bijlenga et al., 2017). As such, the mediation analyses in this study were both statistically and theoretically justified.

With this study, we aimed to assess whether variability in orientation discrimination performance is differentially related to ADHD and ASD traits. First, we hypothesized that higher levels of these traits would be associated with altered sensory sensitivity. Specifically, if the findings of Dickinson et al. (2014) are correct, we expected that increased trait levels would be associated with enhanced performance on the orientation task, however such examination has not been applied to ADHD. However, given the conflicting evidence in the literature, this aspect of the study also has an exploratory component. Second, we hypothesized that anxiety would mediate the relationship between ADHD/ASD traits and sensory thresholds, based on its established role in modulating sensory and attentional processing in both conditions. Third, we expected these patterns to apply similarly to ADHD and ASD traits, given prior work suggesting commonalities in their cognitive and sensory profiles. We used mediation analysis to establish the potential intermediary role of anxiety. These questions were investigated in a sample of adults with dimensionally distributed ADHD and ASD traits, consistent with evidence that psychiatric traits lie on a continuum, with the extreme end of the spectrum warranting clinical diagnosis. Such dimensional models have long been supported by genetic research (DeFries and Fulker, 1985; Levy et al., 1997).

## Methods

### Participants

One hundred and thirty-six participants were initially recruited through student and staff support groups at the University of Sheffield and the wider Sheffield area. All participants had normal or corrected-to-normal vision. Data from thirty three participants were excluded from the final analysis because they did not complete the full set of questionnaires administered via Qualtrics, meaning their responses could not be processed. This resulted in a final sample of one hundred and three participants who completed all sections of the study. Of these, fifty nine identified as female, forty as male, and four as non-binary. The majority (60%) identified as White European/British/Irish with a college education, while the remaining 40% represented a mix of ethnic backgrounds, including Asian, Black, and Mixed Ethnicity. Participants ranged in age from 18 to 57 years (*M* = 22.68, *SD* = 7.68). All participants provided informed consent and received detailed information and debrief forms in accordance with the Declaration of Helsinki (World Medical Association, 2013). Ethical approval was granted by the university's Ethics Committee. Each participant was assigned a unique, non-identifiable code to ensure confidentiality.

### Questionnaires

The study employed scales developed specifically for testing dimensional psychiatric disorders within the adult population. All responses were collected via the online survey system Qualtrics XM (Qualtrics, U.S.A.), and were presented in a randomized order. For ADHD traits the Adult ADHD Self Report Scale (ASRS) was used (Kessler et al., 2005). For ASD traits, The Broad Autism Phenotype Predict scale (BAPQ) (Hurley et al., 2007) was used. To measure panic and social anxiety we employed Screen for Adult Anxiety Related Disorders (SCAARED) (Angulo et al., 2017) and for generalized anxiety we used the Generalized Anxiety Disorder scale (GAD—Spitzer et al., 2006). Finally, to compare self-reported sensory issues with our psychophysical measures, we used the Glasgow Sensory Questionnaire (Robertson and Simmons, 2019).

The Adult ADHD Self Report Scale (ASRS; Kessler et al., 2005) is an eighteen item scale based on the DSM-IV criteria for ADHD, measuring the Inattention (IN) and hyperactivity/impulsivity (HP) traits. A Likert scale ranging from Never-Rarely-Sometimes-Often-Very Often is used to rate how much each statement applies to the respondent considering everyday life in the past 6 months. The ASRS has a two-factor structure which includes an Inattention scale and a Hyperactivity/impulsivity scale. Each subscale contains nine items (e.g., Inattention: “How often do you have problems remembering appointments or obligations?”, Hyperactivity/impulsivity: “How often do you interrupt others when they are busy?”).

The Broad Autism Phenotype Questionnaire (BAPQ; Hurley et al., 2007) is a thirty six item scale consisting of 3 subscales—Aloof Personality (AP), Pragmatic Language (PL), and Rigid Behavior (RB). Respondents to rate their behavior for the past 6months using Very Rarely-Rarely-Occasionally-Somewhat Often-Often-Very Often Likert scale Each subscale contains thirteen items (e.g., AP: “I would rather talk to people to get information than to socialize”; PL: “It's hard for me to avoid getting sidetracked in conversations”; RB: “I am comfortable with unexpected changes in plans”).

The Panic/Somatic and Social anxiety scales from the SCAARED questionnaire (Angulo et al., 2017) were used to measure the respective constructs in the cohort. Respondents rated how much the statements provided in the questionnaire matched their behavior by choosing between Not True- Somehow True-Very true, to scale items such as “I get shaky” (Panic/Somatic) or “I feel nervous with people I don't know well” (Social).

The Generalized Anxiety Disorder scale (Spitzer et al., 2006) is a 7 item scale which asks participants to reflect on behavior from the last 2 weeks and rate themselves on statements such as “I feel nervous, anxious or on edge” with Not at all—Several days—More than half the days—Nearly everyday.

The Glasgow Sensory Questionnaire (GSQ; Robertson and Simmons, 2019) measures self-rated hyper—and hypo-sensory sensitivity to stimuli across all sensory modalities—visual, auditory, gustatory, olfactory, tactile vestibular, and proprioception across forty two items (Varbanov et al., 2023; Robertson and Simmons, 2019). Statements such as “Do you find certain noises/pitches of sounds annoying” are responded to with Never-Rarely-Sometimes-Often—Always. Respondents are advised to think of ordinary activities in everyday life rather than exceptional situations.

### Visual orientation discrimination task

**Figure 1:**
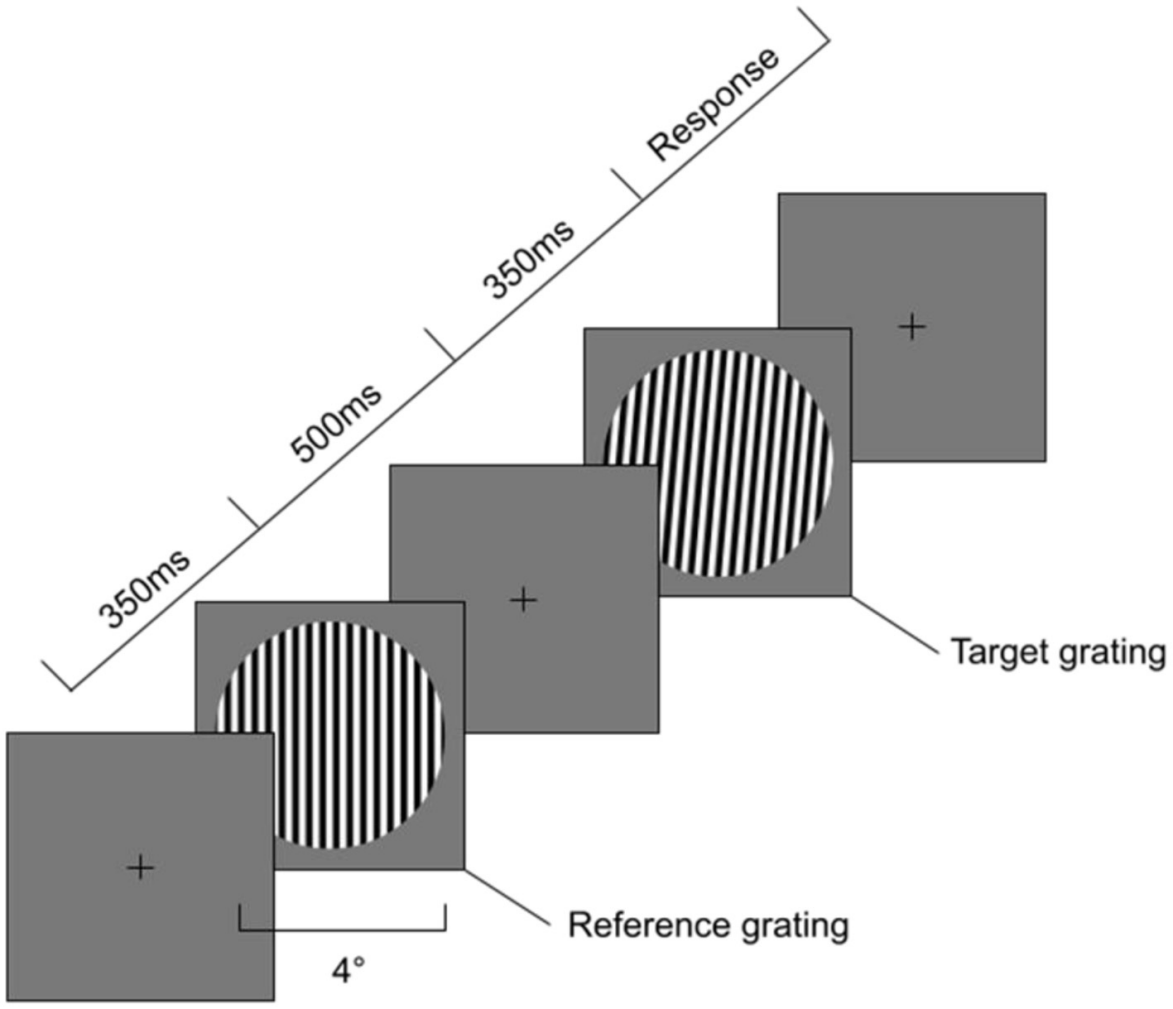
Sequence of events during Orientation Discrimination task. First a 350 ms Reference grating is presented at either vertical or oblique angle with a diameter of 4 degrees visual angle. Second an Interstimulus Interval with a fixation cross for 500ms is presented, followed by a Target grating a number of degrees away from the Reference grating in either clockwise or anti-clockwise rotation.

To measure visual orientation discrimination thresholds we used a method of constant stimuli with a two-alternative, forced choice, adaptive, randomly interleaved staircase procedure with a one-up-three-down design based on that used by Dickinson et al. (2014) and Edden et al. (2009). Participants were presented with a reference sinusoidal grating, followed by a target grating, both visible for 350 ms and separated by a 500 ms interstimulus interval. Their task was to identify whether the target grating was rotated clockwise or anticlockwise relative to the reference grating (using the left [for anticlockwise] and right [for clockwise] arrow keys). The sequence of presentations on each trial can be found in Figure 1. The design was created using PsychoPy v. 2022.2.4. The (Oblique or Vertical) Reference + Target pair were mixed so participants never saw only Oblique pairs or only Vertical pairs. Each grating had the following parameters—diameter 4cm, spatial frequency three cycles, mean luminance 45 cd/m2 and 80% contrast. A linearised LCD monitor was used with a circular aperture attached to it in order to prevent orientation cues coming from the edges of the monitor. A chinrest placed 57 cm away from the monitor was used to stabilize the head of the participant, meaning that the grating occupied 4 degrees of visual angle. Gamma and monitor luminosity were calibrated using the Gamma Calibration settings in PsychoPy and a photometer. The target grating always started at 5 degrees relative to the reference grating. The difference between the reference and target orientation then decreased (making the task harder) following three correct responses and increased (making the task easier) following one incorrect response until 9 reversals were reached. On each reversal, the step size for the orientation increment/decrement changed to 75% of the previous. The maximum value for the target grating orientation was 20 degrees relative to the reference, with a minimum of 0.001 degrees relative to the reference grating. The task consisted of two conditions—a vertical (where the reference grating was oriented at 0 degrees) and an oblique (where the reference grating was oriented at 45 degrees angle). Two staircases reflecting the clockwise and anticlockwise changes were used for each condition. Therefore, participants were presented with four staircases (or types of gratings) —at 0 clockwise, 0 anticlockwise, 45 clockwise and 45 anticlockwise. Staircases converged at 79% correct performance (Leek, 2001).

### Procedure

An information sheet was provided and consent taken from all participants prior to commencing the study. Participants completed the visual task first, followed by the battery of questionnaires on two different computers within the same testing space. They were asked to store any light producing equipment away and stayed in the testing room for 30 m prior to commencing the visual task. In this time all light producing devices were switched off and cover equipment placed in front of the monitors in order to allow dark adaptation (Kalloniatis and Luu, 1995). The visual task was divided in two main sections—a practice section, followed by a 40 s break and the main task session, split into blocks with a 2 min break in between, lasting approximately 17 min in total. Once completed, participants were seated on another computer where they filled the battery of questionnaires described above. Upon completion, participants were debriefed.

## Data analysis

### Power analysis

Based on Fritz and MacKinnon (2007), a power analysis for a medium effect size (with α = 0.05 and power = 0.80) indicated that seventy one participants were needed for our primary mediation analyses. As we included one hundred and three participants our analysis, we observed that the *a* and *b* paths indeed fell within the medium-to-large range, supporting the contention that our sample size was sufficient to detect the indirect effects. To further enhance the robustness of our effect estimates, we employed bias-corrected bootstrap procedure (with 5,000 resamples) for all mediation models, in order to generate more accurate confidence intervals for the effects.

### Averaging the thresholds

To find the visual discrimination task thresholds, we discarded the first two reversals due to learning effects and used the remaining seven reversals to calculate the mean thresholds. Averaged thresholds were calculated across both sections of the main task for each participant individually. The averaged thresholds were established by calculating the mean of the values at the point of reversal for each staircase on both conditions—vertical and oblique. To estimate the total means for the cohort, the mean of each condition and each staircase was taken and averaged across both runs.

### Correlations

Internal consistency was assessed using Cronbach's alpha. The ASRS showed excellent internal reliability (α = 0.91), with subscales ADHD-IN and ADHD-HP both at α = 0.86. The BAPQ total and its subscales all had acceptable reliability (α = 0.70). SCAARED subscales for panic and social anxiety showed α = 0.89 and α = 0.90 respectively. The GAD scale had α = 0.85, and the GSQ showed excellent reliability at α = 0.94.

Total scale and subsequent subscale scores were calculated for all questionnaires within the battery. Following the reliability (α > 0.7) analysis, the distributional properties of the data were evaluated using the Shapiro-Wilk test. Subsequently, correlations explored the relationships between the overall ADHD/ASD scale scores and the task thresholds. Individual subscales and their relationship with the task thresholds were subsequently explored as was the potential involvement of anxiety in those relationships. These were then corrected for multiple comparisons using Benjamini-Hochberg FDR correction (*q* = 0.05).

### Mediation analysis rationale

To determine whether anxiety mediates the relationship between ADHD/ASD traits and sensory thresholds, we conducted mediation analyses using Model 4 of Hayes' PROCESS macro (version 4.2) in IBM SPSS version 26 (Hayes, 2022). This approach is based on a series of Ordinary Least Squares (OLS) regression models that estimate the total effect of the predictor on the outcome (path c), the effect of the predictor on the mediator (path a), the effect of the mediator on the outcome controlling for the predictor (path b), and the direct effect of the predictor on the outcome controlling for the mediator (path c′). Mediation was tested only for variable sets where significant correlations were found between all three components (predictor, mediator, outcome) following FDR correction (*q* = 0.05). Standardized scores were reported for all mediation models. Indirect effects were assessed using a bias-corrected bootstrap method with 5,000 resamples to generate 95% confidence intervals. An indirect effect was considered statistically significant if the bootstrap confidence interval did not include zero. In addition to estimating the size of the indirect effect, total and direct effects were also reported to distinguish between partial and full mediation.

## Results

### Descriptive statistics

To evaluate the clinical relevance of participants' self-reported trait scores, we compared scores on the BAPQ and ASRS against established clinical thresholds. For the BAPQ subscales, a score above 3.25 on Aloof Personality (AP) indicates potential clinical relevance; eigth out of 103 participants (84.5%) exceeded this threshold, with a mean AP score of 3.68 (*SD* = 0.53). For Rigid Behavior (RB), thirty two participants (31.1%) scored above the clinical threshold of 3.65, with a mean of 3.36 (*SD* = 0.50). For Pragmatic Language (PL), seventy six participants (73.8%) scored above the threshold of 2.50, with a mean of 3.16 (SD = 0.69) (Hurley et al., 2007). Regarding ADHD traits, approximately 60 participants (58.3%) scored above the clinical cutoff of 14 on the ASRS Part A, with a mean ASRS-A score of 13.55 (SD = 4.50) (Kessler et al., 2005). These figures suggest that a substantial portion of the sample endorsed trait levels within or approaching clinical ranges.

### Data and participant characteristics

The Shapiro-Wilk test indicated that all variables were normally distributed, except Panic and Social anxiety. Therefore Spearman (rho) are used for Panic/Social anxiety and Pearson correlations (r) are used for the other variables in the analyses below. In terms of the characteristics of the participant sample (see Table 1), outcomes for the ASD subscales indicate a mean for the AP subscale of 44.21 out of max. 59 (SD 6.40) followed by the RB subscale with a mean of 40.37 out of 56 max., (SD 5.96) and PL subscale with a mean of 38 out of 56 max., and SD 8.26. The two ADHD subscales- inattentive traits (mean of 22.26 out of max., 36, SD 6.80) and hyperactivity/impulsivity (mean of 18.36, SD 7.49) suggest typical division of symptoms for an adult cohort with higher inattentive than hyperactive traits. The cut off provided by the authors of the scale indicate significant symptoms of hyperactivity/impulsivity (< 14) and just below threshold inattentive traits (< 24). The highest score of the anxiety scale is for panic anxiety (mean 29.76, out of max 47 with SD 7.91), followed by GAD (mean of 15.25, out of max., 28, SD 5.10) and social anxiety with mean of 13.72 out of max 21, SD 4.38. A cut off score of 15 or above on the GAD scale indicates severe levels of anxiety. The sensory scale showed a wide range of sensory experiences with a mean of 102.95, SD 26.28.

**Table 1:** Descriptive statistics for the self-reporting scales for the 103 participants presenting Minimum, Maximum, Mean and Standard Deviation values.

**Scale & subscales**	**Minimum**	**Maximum**	**Mean**	**Std. deviation**
**Visual orientation discrimination thresholds**				
Vertical	0.454	7.717	1.663	0.99
Oblique	0.244	12.16	3.648	2.28
**Glasgow Sensory Questionnaire (GSQ)**	56	180	102.95	26.28
**Screen for Child Anxiety Related Emotional Disorders—Adult (SCAARED)**				
Panic	17	47	29.76	7.911
Social	7	21	13.72	4.376
**Generalized Anxiety Disorder Scale (GAD)**	0	28	15.25	5.095
**Adult ADHD Self-Report Scale (ASRS)**	12	69	40.62	13.157
Inattention (IN)	7	36	22.26	6.804
Hyperactivity—Impulsivity (HP)	2	36	18.36	7.488
**Broad Autism Phenotype Questionnaire (BAPQ)**	71	185	121.32	25.517
Aloof Personality (AP)	30	59	44.21	6.369
Rigid Behavior (RB)	28	56	40.37	5.956
Pragmatic Language (PL)	20	56	38	8.256

GSQ, Glasgow Sensory Questionnaire; SCAARED, Screen for Child Anxiety Related Emotional Disorders – Adult version; GAD, Generalized Anxiety Disorder Scale; ASRS, Adult ADHD Self-Report Scale; BAPQ, Broad Autism Phenotype Questionnaire.

### Significance of the sensory thresholds findings

The orientation discrimination results show a clear and significant difference between the oblique and the vertical thresholds [t (103) = −9.156, *p* < 0.0001]. The mean score for the oblique threshold, standing at 3.65, was significantly higher than that for vertical, standing at 1.66, with a mean difference of −1.985 (95% *CI:* −2.415 to−1.555, *SD* = 2.211). Therefore, the oblique threshold was higher by a factor of 2.19 ± 1.0. These results show a consistent and substantial significant difference throughout the cohort with higher oblique and lower vertical thresholds, confirming a classical oblique effect.

### Relationship between the sensory thresholds and the self reported traits

Table 2 shows a significant positive correlation between the oblique threshold and the overall scores on the GSQ (*r* = 0.221, *p* < = 0.5). However, it is noteworthy that the same positive and significant relationship is not observed for the vertical threshold (*r* = 0.047, *p* > 0.05). ASRS and BAPQ total scores correlate positively and significantly with both the vertical and oblique thresholds— *r* = 0.285, *p* = 0.004 for ADHD/vertical; *r* = 0.239, *p* = 0.05 for ADHD/oblique; *r* = 0.272, *p* < 0.005 for ASD/vertical; *r* = 0.286, *p* < 0.5 for ASD/oblique. These results suggest that higher levels of ADHD and ASD traits are associated with worse instead of better performance on the task. To further examine which specific ADHD/ASD subtraits might drive these effects, Table 3 presents correlations between sensory thresholds and individual subscales. Among the ADHD traits, the Hyperactivity-Impulsivity (HP) subscale showed significant positive correlations with both vertical (*r* = 0.347, *p* = 0.001) and oblique (*r* = 0.246, *p* = 0.012) thresholds. The Inattention (IN) subscale did not correlate significantly with vertical thresholds and showed only a marginal relationship with oblique thresholds (*r* = 0.191, *p* = 0.054). For ASD traits, the Aloof Personality (AP) subscale correlated with vertical thresholds (*r* = 0.314, *p* = 0.01), while the Pragmatic Language (PL) subscale was significantly related to oblique thresholds *(r* = 0.301, *p* = 0.002). The Rigid Behavior (RB) subscale did not correlate significantly with either threshold. Correlations between ADHD and ASD subscales were generally low, with the exception of ADHD-HP and ASD-RB (*r* = 0.315, *p* = 0.01), suggesting limited overlap between dimensions.

**Table 2:** Correlational analysis on overall scores for each scale.

**Variables**	**Vertical**	**Oblique**	**Sensory**	**Panic**	**Social**	**GAD**	**ASRS total**	**BAPQ total**
Vertical	1							
Oblique	0.29^**^	1						
Sensory	0.05	0.22^*^	1					
Panic	0.25^**^	0.29^**^	0.68^**^	1				
Social	−0.14	0.12	0.42^**^	0.42^**^	1			
GAD	0.19^*^	0.30^**^	0.55^**^	0.58^**^	0.40^**^	1		
ASRS_total	0.28^**^	0.24^*^	0.48^**^	0.37^**^	0.12	0.52^**^	1	
BAPQ_total	0.27^**^	0.29^**^	0.19	0.19^*^	−0.12	0.07	0.19	1

Pearson correlations were used for all scales except Panic and Social anxiety, where Spearman correlations were estimated. ASRS_total = total scores for the Adult ADHD Rating Scale; BAPQ_total = total scores for the Broad Autism Phenotype Questionnaire.

^**^Correlation is significant at the 0.01 level (2-tailed).

^*^Correlation is significant at the 0.05 level (2-tailed).

**Table 3:** Correlational analysis on remaining scales of specific traits.

**Variables**	**Vertical**	**Oblique**	**Sensory**	**Panic**	**GAD**	**IN**	**HP**	**AP**	**RB**	**PL**
Vertical	1									
Oblique	0.29^**^	1								
Sensory	0.05	0.22^*^	1							
Panic	0.25^**^	0.29^**^	0.68^**^	1						
GAD	0.19^*^	0.30^**^	0.55^**^	0.58^**^	1					
IN	0.17	0.19	0.36^**^	0.27^**^	0.43^**^	1				
HP	0.35^**^	0.25^*^	0.52^**^	0.43^**^	0.53^**^	0.69^**^	1			
AP	0.31^**^	−0.02	−0.26^**^	−0.16	−0.08	0.03	0.10	1		
RB	0.12	0.16	0.56^**^	0.30^**^	0.25^*^	0.14	0.31^**^	−0.18	1	
PL	0.05	0.30^**^	0.07	0.17	−0.02	−0.01	0.04	0.04	−0.01	1

Pearson correlations were used for all scales except Panic and Social anxiety, where Spearman correlations were estimated. Sensory, Self-reported sensory traits; Panic, Panic anxiety; Social, Social anxiety; GAD, Generalized Anxiety Disorder scale; IN, Inattention traits; HP, Hyperactivity traits; AP, Aloof Personality traits; RB, Rigid Behavior traits; PL, pragmatic Language traits.

^**^Correlation is significant at the 0.01 level (2-tailed).

^*^Correlation is significant at the 0.05 level (2-tailed).

Among the three types of anxiety measured, panic anxiety correlated significantly with both vertical *(r* = 0.254) and oblique (*r* = 0.291) thresholds (both *p* ≤ 0.01), indicating that higher anxiety levels are associated with increased sensory thresholds. Of the three candidate traits initially considered for mediation analysis (ADHD-HP, ASD-AP, and ASD-PL), panic anxiety correlated significantly only with ADHD-HP (*r* = 0.429, *p* < 0.01). Generalized anxiety disorder (GAD) scores also correlated with both thresholds—vertical: *r* = 0.194, *p* = 0.050; oblique: *r* = 0.299, *p* ≤ 0.01,—and with ADHD-HP (*r* = 0.527, *p* < 0.01).

Based on FDR-corrected correlations, the ADHD-HP subscale remained significantly associated with both vertical (*r* = 0.347, *p* < 0.01) and oblique (*r* = 0.246, *p* < 0.05) thresholds. Panic anxiety also maintained significant correlations with both thresholds (vertical: *r* = 0.254; oblique: *r* = 0.291, both *p* < 0.01), as well as with ADHD-HP (*r* = 0.429, *p* < 0.01). GAD was significantly related to ADHD-HP (*r* = 0.527, *p* < 0.01) and to the oblique threshold (*r* = 0.299, *p* < 0.01), but not to the vertical threshold (*r* = 0.194, *p* = n.s.). Although ASD traits such as AP and PL showed selective associations with the thresholds (*r* = 0.314 and *r* = 0.301, respectively), they were excluded from mediation modeling because they did not significantly correlate with either anxiety variable following FDR correction.

Accordingly, three models were selected for further analysis based on the pattern of FDR-corrected correlations: one examining the associations among oblique thresholds, panic anxiety, and ADHD-HP; a second model involving oblique thresholds, GAD, and ADHD-HP; and a third model focusing on vertical thresholds, panic anxiety, and ADHD-HP. These models include only variables for which all pairwise associations—between thresholds, anxiety symptoms, and ADHD-HP—remained statistically significant following correction for multiple comparisons.

### Exploring the potential mediating role of anxiety between sensory thresholds and self-reported ADHD traits

**Figure 2:**
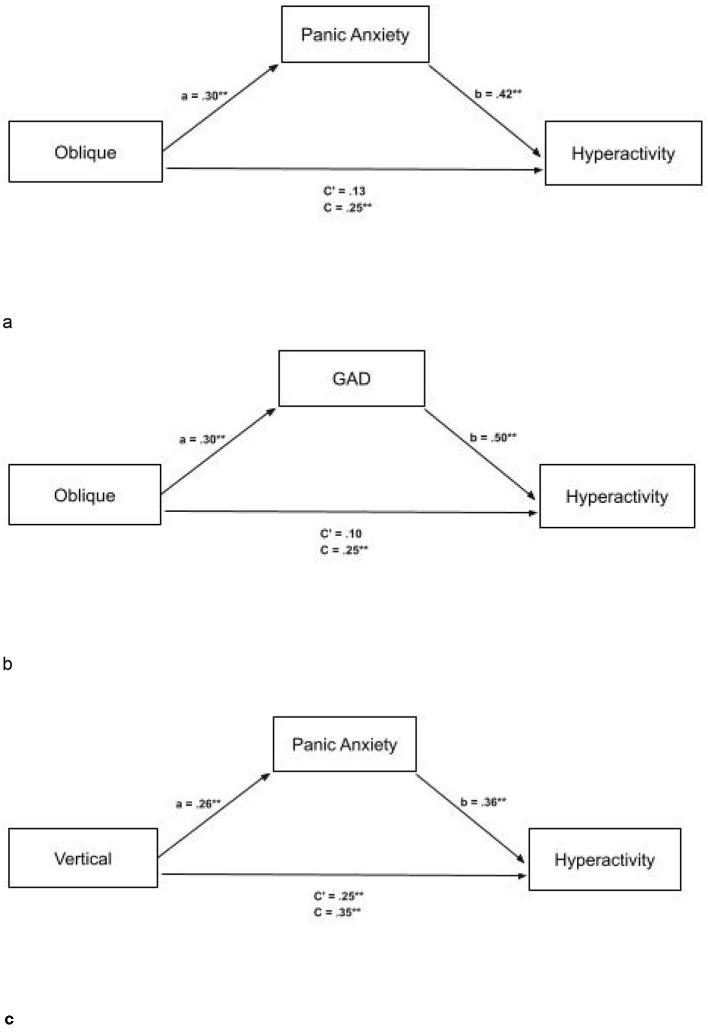
**(a)** Full mediation between oblique thresholds and ASRS-HP (Hyperactive traits) with mediator panic anxiety with standardized scores. The mediator fully explains the relationship between oblique thresholds and ASRS-HP. ASRS, Adult ADHD Rating Scale. **(b)** Full mediation between oblique thresholds and ASRS-HP (Hyperactive traits), with mediator GAD with standardized scores. The mediator fully explains the relationship between oblique thresholds and ASRS-HP. ASRS, Adult ADHD Rating Scale. **(c)** Partial mediation between vertical thresholds and ASRS-HP (Hyperactive traits), with mediator Panic Anxiety with standardized scores. The mediator partially explains the relationship between vertical thresholds and ASRS-HP. ASRS, Adult ADHD Rating Scale. *p < 0.5, **p < 0.1.

We examined the association between oblique orientation discrimination thresholds and ADHD hyperactivity–impulsivity (ADHD-HP) scores, with panic anxiety included as a potential intervening variable (see Figure 2a). Standardized regression coefficients showed that higher oblique thresholds were associated with greater panic anxiety (a path: β = 0.30, *p* = 0.002). Panic anxiety was positively associated with higher ADHD-HP scores in a simple regression (β = 0.42, *p* < 0.001), and this association remained significant when controlling for oblique thresholds (b path: β = 0.38, *p* < 0.001). The total standardized association between oblique thresholds and ADHD-HP (c path) was β = 0.25 *(p* = 0.012). When both oblique thresholds and panic anxiety were entered into the model, the direct association (c′ path) decreased to β = 0.13 (*p* = 0.167). The indirect effect of oblique thresholds on ADHD-HP scores through panic anxiety was statistically significant, with a bias-corrected 95% bootstrap confidence interval of [0.14, 0.74].

We also examined the association between oblique orientation discrimination thresholds and ADHD hyperactivity–impulsivity (ADHD-HP) scores, with Generalized Anxiety Disorder (GAD) symptoms included as a potential intervening variable (see Figure 2b). Standardized regression coefficients indicated that higher oblique thresholds were associated with greater GAD symptoms (a path: β = 0.30, *p* = 0.002). GAD symptoms were positively associated with higher ADHD-HP scores in a simple regression (β = 0.50, *p* < 0.001), and this association remained significant when controlling for oblique thresholds (b path: β = 0.50, *p* < 0.001). The total standardized association between oblique thresholds and ADHD-HP (c path) was β = 0.25 *(p* = 0.012). When both oblique thresholds and GAD symptoms were entered into the model, the direct association (c′ path) decreased to β = 0.10 (*p* = 0.278). The indirect effect of oblique thresholds on ADHD-HP scores through GAD symptoms was statistically significant, with a bias-corrected 95% bootstrap confidence interval of [0.20, 0.89].

We further explored the association between vertical orientation discrimination thresholds and ADHD hyperactivity—impulsivity (ADHD-HP) scores, with panic anxiety included as a potential intervening variable (see Figure 2c). Standardized regression coefficients indicated that higher vertical thresholds were associated with increased panic anxiety (a path: β = 0.26, *p* = 0.007). Panic anxiety was positively associated with ADHD-HP scores in a simple regression and remained significant when controlling for vertical thresholds (b path: β = 0.36, *p* < 0.001). The total standardized association between vertical thresholds and ADHD-HP (c path) was β = 0.35 (*p* < 0.001). When panic anxiety was included in the model, the direct effect (c′ path) was reduced to β = 0.25 (*p* = 0.006). The indirect effect of vertical thresholds on ADHD-HP through panic anxiety was statistically significant, with a bias-corrected 95% bootstrap confidence interval of [0.27, 1.55].

## Discussion

This study was largely influenced by a fundamental, yet unanswered question in the literature on ADHD and ASD- are they a common overarching disorder as claimed by Hayashi et al. (2022) or two distinct conditions? As discussed at the beginning of this work, although ADHD and ASD present with different symptomatology, from a sensory processing perspective they seem to have more in common than not. However, previous work (Varbanov et al., 2023) has suggested some commonality but overall a separation between ADHD and ASD traits aided by other comorbidities and no direct connection with sensory processing but rather a third intermediary factor acting as a connector and modulator- that of panic anxiety. These results raise further questions whether it is possible that comorbid conditions have a much bigger role in the presentation of ADHD/ASD traits and their connection with sensory impairments than previously thought. However, omissions in previous research on low level processing with regards to ADHD and research primarily focused on ASD groups have made it difficult to answer such questions. In addition, previous studies have largely been based on self- reporting, and this bears its own risks with socially desirable responding. Indeed, scores on the Glasgow Sensory Scale in the present study only correlated significantly with oblique thresholds, thus validating our shift to a more robust psychophysical approach to assessing sensory function here.

Although this study was conducted in a non-clinical adult sample, investigating dimensional traits of ADHD and ASD offers several methodological advantages. Studying subclinical populations allows researchers to examine variation in trait expression across a broader and more continuous spectrum, avoiding diagnostic thresholds that can obscure subtle effects (Constantino and Todd, 2003). This dimensional approach is also more statistically powerful for detecting associations with other psychological variables, such as anxiety or sensory thresholds, and avoids the confounds introduced by medication, clinical comorbidities, or heterogeneous diagnostic criteria that often complicate clinical samples (Lubke et al., 2009). Furthermore, research suggests that many cognitive and perceptual differences seen in ADHD and ASD exist on a continuum within the general population (Robinson et al., 2013), supporting the validity of this approach. As such, our findings reflect trait-level associations in a typically developing cohort, and future research is encouraged to examine whether these associations generalize to clinical samples.

### Increase in severity of traits associated with worse task performance

First, this study found significant positive correlations between the ADHD and ASD total scores, and the task thresholds (Davis and Plaisted-Grant, 2015). The positive correlations indicated that as the levels of the core ADHD/ASD self-reported traits increased in severity, the performance on the visual task worsened with more incorrect responses being given on both the vertical and oblique staircases. These findings indicate a common impaired visual sensory functioning between ADHD and ASD and are hence supported by previous studies suggesting that it is possible that both conditions overlap on sensory impairments and exacerbate each other's sensory behavior and symptoms (Rao and Landa, 2014). The findings are further supported by research suggesting high levels of visual dysfunction in ADHD/ASD (Sanz-Cervera et al., 2017).

These results are somewhat surprising but not unusual for ASD, as despite previous research (Dickinson et al., 2014; Bertone et al., 2005) reporting visual superiorities, a voluminous body of work indicates otherwise. Happé and Frith (2006), who report that severity of detail-focused cognitive styles related to weak central coherence can impact visual processing, support our findings. In addition, Brock et al. (2011) reported no enhanced performance attributed to higher ASD levels on discrimination tasks in subclinical populations and instead suggested that factors such as processing speed, attentional control, working memory, or other executive functions affecting visual search may prove a better explanation for ASD advantage in performance. In line with this, reports in children with ASD did not support significant advantages in orientation discrimination performance and concluded that potential enhanced perceptual functioning in ASD may not generalize to all types of low-level visual tasks, particularly those involving orientation processing (Manning et al., 2015). Both these studies suggested that impairments in broader cognitive processes that build upon perceptual input—such as integrating information into a coherent whole, flexibly shifting attention, and applying reasoning or problem-solving strategies—could affect the processing of incoming information through factors like weak central coherence (Happé and Frith, 2006) or hyper-systematizing (among other factors—Baron-Cohen et al., 2009) and thus result in seemingly enhanced sensory performance. Such performance, however, would not be due to the simple enhancement of low level sensory processing. These studies are further supported by a report from Simmons et al. (2009) who conducted an extensive review on visual processing in ASD and concluded that findings thus far are mixed and in fact some studies in their review report reduced rather than enhanced performance in tasks involving orientation discrimination. In conjunction with our findings, the above offer a more nuanced picture of visual behavior in ASD and highlight that the previously reported strict visual superiority over ADHD should be evaluated within the context of other factors, including impairments in higher order cognition and the possible interaction between ASD and other comorbidities, such as ADHD, which can further impair behavior (Rao and Landa, 2014).

Sensory—motor integration, particularly as modulated by the cerebellum, may also contribute to the observed sensory processing patterns in ADHD and ASD. The cerebellum is not only essential for fine-tuning motor control but also for coordinating sensory input with motor responses, influencing timing, prediction, and error correction across modalities (Ivry and Spencer, 2004; Baumann et al., 2015). Altered cerebellar function, reported in both ADHD and ASD (Stoodley, 2016), could disrupt these integrative processes, potentially amplifying perceptual inefficiencies and contributing to the impaired orientation discrimination performance observed in the current study.

Although the prevailing narrative often links ASD with hypersensitivity, our findings may reflect an alternative mechanism, particularly in non-clinical or undiagnosed individuals with high trait expression. A substantial proportion of our sample scored above clinical thresholds on the BAPQ subscales, suggesting that reduced sensitivity was not simply driven by low trait expression. Rather, it is possible that in individuals with high but undiagnosed ASD traits, sensory atypicalities manifest differently than in clinically diagnosed populations. Reduced sensitivity in orientation discrimination may reflect atypical sensory encoding, diminished neural gain, or attentional filtering strategies developed to manage sensory load. These mechanisms could dampen perceptual precision rather than amplify it, especially under task demands requiring fine-grained visual discrimination. Additionally, subclinical populations might exhibit compensatory adaptations or altered perceptual priorities, where sensory input is deprioritised in favor of top-down control. This highlights the need to interpret sensory findings in ASD not only through the lens of hypersensitivity but within the broader context of individual variation in trait expression, coping mechanisms, and comorbidity profiles.

On the other hand, ADHD studies on inefficient attention deployment such as inability to sustain attention or inefficient attention shifting in visual tasks, support the notion of exacerbated hyperactivity which can lead to more distracted and restless behavior and thus worse performance (Fabio and Urso, 2014; Canu et al., 2022). These reports align with our results as the total ADHD scores did correlate positively with the thresholds, suggesting higher level of mistakes associated with increased levels of ADHD traits. In addition they align with the results from the second level of analysis which showed that scores on the inattention and hyperactivity subscales of the ASRS correlated significantly with each other. Further, as ADHD is associated with working memory deficits and slower processing speed—both of which can compromise efficiency in visual tasks—a study by Canu et al. (2022) suggested that delay in initiating visual search is characteristic of ADHD and results in worse performance on tasks where hardly distinguishable items are searched for. Reduced processing speed may limit the rapid comparison of visual stimuli, especially as task difficulty increases, and could interact with impaired top-down control of attention to exacerbate performance difficulties (Valmiki et al., 2021). It is important to underline though that there is scarce research on low-level visual perception in ADHD and more is needed to establish the exact mechanisms behind the impaired performance observed in the current study. It is possible that, as reported for ASD above, impaired higher order cognition and other comorbidities play a role in ADHD too.

### Differential associations between individual ADHD/ASD traits and visual discrimination

In spite of the support presented above for the impaired visual task performance in ADHD and ASD in our cohort, the similar results reported thus far did not hold when we investigated the individual ADHD and ASD traits as presented in Table 3. Looking at the individual traits, we found that only the hyperactive ADHD traits correlated significantly with both conditions on the sensory task, whilst the ASD subscales correlated either with the vertical (for AP) or oblique thresholds (for PL). These specific correlations suggest that the afore discussed common sensory atypicality/impairment with regards to visual discrimination in the two conditions might instead come from more targeted pathways of interaction with sensory effects in ADHD and ASD. As suggested by previous reports, these more targeted pathways could include the role of comorbidities (such as anxiety) as it was found that the ADHD/ASD traits and sensory processing traits, albeit in two separate clusters, were influenced in the expression and connection of their symptoms by anxiety. This interaction manifested in similar sensory and ADHD/ASD associated impaired behavior for both groups.

The above falls in line with the results of both the full and partial meditations we found, as they suggest that anxiety either completely or partially accounts for the relationship between the sensory thresholds and the hyperactive ADHD traits - none of the ASD traits related to the sensory thresholds in a similar manner. This difference in mediations suggests, as discussed above, a more specific link (a targeted pathway or interaction) between ADHD hyperactivity and generalized or panic anxiety which does not relate similarly to the ASD traits. It is possible that the effect of this interaction contributes to the impaired performance on the visual task for the ADHD group, although more research is needed to confirm how this comes about. In support of this idea, van der Meer et al. (2018) reported that increased panic/generalized anxiety in ADHD worsened performance on a visuospatial working memory task as high levels of anxiety had adverse effects on performance because irrelevant thoughts interfered with information processing [cognitive interference theory—(Barkley, 1997)]. They further reported that due to the interaction between ADHD and anxiety with working memory capacity, reduced performance on the visuospatial tasks was observed and suggested aberrant dorsolateral prefrontal cortex activity due to the cumulative interaction of anxiety and ADHD traits. They concluded that this effect cannot be attributed to the additive effects of ADHD and anxiety but to a unique interaction between the two. Although van der Meer did not investigate the individual symptoms of ADHD, others have found that anxiety can have an effect on hyperactivity (Michelini et al., 2015; Schatz and Rostain, 2006). Although our study did not find significant interactions in relation to ADHD-inattention, Michelini et al. (2015) suggest that increased anxiety can affect cognitive load and increase distractibility and difficulty with concentration, therefore exacerbating inattentive symptoms and leading to restlessness and fidgeting (two symptoms within the hyperactivity/impulsivity dimension). More research is needed to disambiguate the exact role of anxiety, however these studies and our results suggest a much closer interaction negatively affecting sensory behavior than previously considered.

We would argue that anxiety is not unique in its role as a potential mediator and neither is its relationship with the hyperactive traits as other comorbidities are likely to form different (or similar) pathways of interactions with the core ADHD/ASD traits and affect task performance and behavior differently. It is therefore reasonable to suggest that the lack of significant correlations and mediations in relation to ASD is because they might interact differently with conditions we have not looked at here, however to confirm this further research is needed. These results again come in support for models discussed earlier suggesting that comorbidities *per se* are largely involved in a complex interplay with and between the core ADHD/ASD traits and their interactions with sensory input, thus affecting their similarities and differences (Varbanov et al., 2023). How this interaction comes about and how exactly it influences behavior needs to be investigated further.

The role of anxiety in the fully mediated link between hyperactivity and visual processing found for the oblique condition did not hold for the vertical condition, suggesting that there can be a more direct interaction between visual processing and hyperactivity. This possibility is supported by Jung et al. (2014) who reported that increased hyperactivity in individuals with ADHD might be a compensatory response to sensory processing difficulties. This is so as individuals can experience sensory overload associated with visual information processing and could react with hyperactive behavior such as fidgeting in an attempt to self regulate discomfort. Additional support of our finding of a direct interaction between hyperactivity and visual processing comes from Edden et al. (2012) who reported that reduced GABA concentration lead to deficits in cortical inhibition can lead to behavioral issues such as impulsivity and hyperactivity.

### In regards to the different sensory findings to previous research

Although a vast body of ADHD research is consonant with our findings, the results in relation to ASD are more mixed. In particular, Dickinson et al. (2014), on whose study our own was based, report enhanced performance in ASD. However, they did not account for the possible influence of other comorbidities (such as ADHD) on ASD performance although co-occurrence of ASD with ADHD and other disorders is reported to range from 35% for various types of anxiety (Zaboski and Storch, 2018) to 87% between ADHD and ASD alone (Scandurra et al., 2019; Leitner, 2014; Leyfer et al., 2006). This is important because as discussed above, Rao and Landa (2014) report that the core traits of ADHD/ASD could intertwine and exacerbate each other, thus affecting performance and overall behavior. Also, two further key differences between their study and ours may come to explain the different results. First they did not allow for dark adaptation before commencing the task, which might have been detrimental to the outcome as lack of dark adaptation would result in limited rod activity and reliance on cones which are less sensitive in low light conditions (both tasks were performed on a low illuminated monitor of 83 cd/m^2^ or less) (Kalloniatis and Luu, 1995). Second, Dickinson et al. (2014) used a different questionnaire (the Autism Quotient) which measures constructs such as imagination, social communication and does not align as well with the three core traits of ASD- rigid behavior, aloof personality and pragmatic language as the BAPQ measure we used.

In summary, in this study we found that although ADHD and ASD might have similar levels of sensory processing impairments, as the panic and generalized anxiety constructs were critically involved only in their interaction of ADHD (not ASD) with visual perception, these similar levels of sensory impairments might be affected by or a result of complex interactions between core ADHD/ASD traits and comorbidities. These findings align with some previous studies which suggested that ADHD and ASD are separate from each other and that their sensory expressions are only connected via intermediary conditions.

## Limitations and future directions

This study looked at anxiety due to its key role, which previous research suggested has a substantial contribution to ADHD and ASD. However, other comorbidities should also be examined, as we believe all comorbidities engage in a complex interplay with ADHD/ASD traits to manifest behavior. Factors such as sleep quality and depression were not assessed in the present study, as they were outside our intended scope and have not emerged as relevant modulators in our previous research on ADHD, ASD, and sensory processing (Varbanov et al., 2023). Nevertheless, both sleep disturbance and depressive symptoms can influence cognitive and sensory performance, and their omission means we cannot rule out potential indirect effects on the observed associations. In addition, although self-reports are subject to various biases, such as demand effects, we combine them here with an objective and independent measure of perceptual thresholds, as prior work (Tavassoli et al., 2014) has shown consistent and revealing links between such self-reports and behavioral measures of perceptual and sensory function. Nevertheless, self-reports do remain subject to possible biases. Future research should also concentrate on other sensory modalities, as abnormalities in sensory processing are present across all seven modalities. Other aspects of visual processing should also be explored to better understand if replication of the current results will occur.

## Data Availability

The datasets presented in this study can be found in online repositories. The DOI for accessing the repository can be found below: https://doi.org/10.15131/shef.data.29095214.v1.
